# A prediction model for prognosis of nephrotic syndrome with tuberculosis in intensive care unit patients: a nomogram based on the MIMIC-IV v2.2 database

**DOI:** 10.3389/fmed.2024.1413541

**Published:** 2024-05-30

**Authors:** Shenghua Du, Ning Su, Zhaoxian Yu, Junhong Li, Yingyi Jiang, Limeng Zeng, Jinxing Hu

**Affiliations:** ^1^Department of Nephrology, Guangzhou Chest Hospital, Guangzhou Medical University, Guangdong, China; ^2^Department of Oncology, Guangzhou Chest Hospital, Guangzhou Medical University, Guangdong, China; ^3^State Key Laboratory of Respiratory Disease, Guangzhou Key Laboratory of Tuberculosis, Department of Critical Care Medicine, Guangzhou Chest Hospital, Institute of Tuberculosis, Guangzhou Medical University, Guangdong, China; ^4^State Key Laboratory of Respiratory Disease, Guangzhou Key Laboratory of Tuberculosis Research, Department of Tuberculosis, Guangzhou Chest Hospital, Institute of Tuberculosis, Guangzhou Medical University, Guangdong, China

**Keywords:** intensive care unit, prediction model, Medical Information Mart for Intensive Care IV, nephrotic syndrome, tuberculosis

## Abstract

**Background:**

Currently, a scarcity of prognostic research exists that concentrates on patients with nephrotic syndrome (NS) who also have tuberculosis. The purpose of this study was to assess the in-hospital mortality status of NS patients with tuberculosis, identify crucial risk factors, and create a sturdy prognostic prediction model that can improve disease evaluation and guide clinical decision-making.

**Methods:**

We utilized the Medical Information Mart for Intensive Care IV version 2.2 (MIMIC-IV v2.2) database to include 1,063 patients with NS complicated by TB infection. Confounding factors included demographics, vital signs, laboratory indicators, and comorbidities. The Least Absolute Shrinkage and Selection Operator (LASSO) regression and the diagnostic experiment the receiver operating characteristic (ROC) curve analyses were used to select determinant variables. A nomogram was established by using a logistic regression model. The performance of the nomogram was tested and validated using the concordance index (C-index) of the ROC curve, calibration curves, internal cross-validation, and clinical decision curve analysis.

**Results:**

The cumulative in-hospital mortality rate for patients with NS and TB was 18.7%. A nomogram was created to predict in-hospital mortality, utilizing Alb, Bun, INR, HR, Abp, Resp., Glu, CVD, Sepsis-3, and AKI stage 7 days. The area under the curve of the receiver operating characteristic evaluation was 0.847 (0.812–0.881), with a calibration curve slope of 1.00 (0.83–1.17) and a mean absolute error of 0.013. The cross-validated C-index was 0.860. The decision curves indicated that the patients benefited from this model when the risk threshold was 0.1 and 0.81.

**Conclusion:**

Our clinical prediction model nomogram demonstrated a good predictive ability for in-hospital mortality among patients with NS combined with TB. Therefore, it can aid clinicians in assessing the condition, judging prognosis, and making clinical decisions for such patients.

## Introduction

In recent decades, both nephrotic syndrome (NS) and tuberculosis (TB) have posed significant global public health challenges, imposing substantial health burdens on patients (1, 2). NS is clinically characterized by substantial proteinuria, hypoalbuminemia, severe edema, and hyperlipidemia. Tuberculosis, an infectious disease caused by *Mycobacterium tuberculosis*, has shown significant progress in prevention and treatment over the past few decades. However, it remains one of the leading causes of death from infectious diseases worldwide (3–5).

Traditionally, NS and TB have been treated and studied separately. However, emerging evidence suggests that the coexistence of chronic kidney disease (CKD) and TB is common. The risk of developing TB in patients with CKD is 4–30 times higher than that in individuals with normal kidney function, and the risk increases further as the disease progresses. Dialysis patients have a 6.0–52.5 times higher risk of TB infection than the general population (6), and the risk might be even higher in patients with NS. Most studies have focused on the response to immunosuppressive treatment in NS, kidney outcomes (7–9), and mechanisms and prevention of acute kidney injury (AKI) (10–13), with limited literature on adult NS patients with concurrent TB infection. Hsu et al. (14) observed an incidence rate of infectious complications of 16.8% (17/101) in adult NS patients, primarily pneumonia, cellulitis/fasciitis, and urinary tract infections, with one of the three deaths due to TB pneumonia. In the 5 years following the United Nations High-Level Meeting in 2018, over 7 million people died from TB globally, with an estimated 10.6 million people contracting TB in 2021 (2, 15). The decision to use immunosuppressants in NS patients with TB infection is critical because TB infection, with its lengthy treatment duration, uncertain drug side effects, and resistance issues, may further complicate NS treatment. Patients with NS, owing to impaired immune function and prolonged use of immunosuppressants, are more susceptible to TB infection, which in turn may exacerbate kidney damage and create a vicious cycle. In the past year, we encountered cases of patients with primary nephrotic syndrome who had delayed treatment due to TB infection, two of whom achieved clinical cure through antituberculosis, renal replacement, and prednisone treatment (we are preparing for a case report). However, the overall prognosis of NS complicated by TB has seldom been reported comprehensively.

Currently, several clinical prediction models exist for NS, including age, serum phospholipase A2 receptor antibodies, urine α1-macroglobulin, proteinuria, and age, which predict the progression of membranous nephropathy (16). Age, hematuria, and steroid resistance predicted the progression of childhood NS to CKD with a c-statistic of 0.92 (17). Neutrophil count and quantitative C-reactive protein have shown excellent discriminative ability in predicting bacterial infection in children with recurrent NS, with an AUC of 0.83 (18). Unfortunately, predictive models for TB infection are lacking. Therefore, we utilized data from the Medical Information Mart for Intensive Care IV version 2.2 [MIMIC-IV (v2.2)] database (19) to conduct a cohort study analyzing the in-hospital mortality of NS patients with concurrent TB, identify key risk factors, and develop an effective prognostic prediction model to facilitate disease assessment and clinical decision-making, thereby reducing the incidence of severe NS complicated by TB.

## Materials and methods

### Database introduction

The data in this study were all obtained from the Medical Information Mart for Intensive Care IV version 2.2 (MIMIC-IV v2.2) database, which is the result of a collaboration between Beth Israel Deaconess Medical Center (BIDMC) and the Massachusetts Institute of Technology (MIT). This database encompasses information from the BIDMC for all patients admitted to the emergency department or Intensive Care Unit (ICU) between 2008 and 2019. It records each patient’s duration of hospital stay, laboratory test results, medication treatment, vital signs, and other comprehensive information. To protect patient privacy, all personal information was de-identified with random codes replacing patient identifiers. Therefore, informed consent or ethical approval was not required. The MIMIC-IV (v2.2) database was downloaded from PhysioNet online forum (MIMIC-IV v2.2).^1^ To apply this database for clinical research, the first author of this study, Shenghua Du, completed the Collaborative Institutional Training Initiative (CITI) course and passed the “Conflict of Interest” and “Data or Specimens Only Research” exams (ID: 59379461), signed a data use agreement, and ultimately, our research team was granted the qualification to use this database and extract data.

### Patient selection criteria

**Figure 1:**
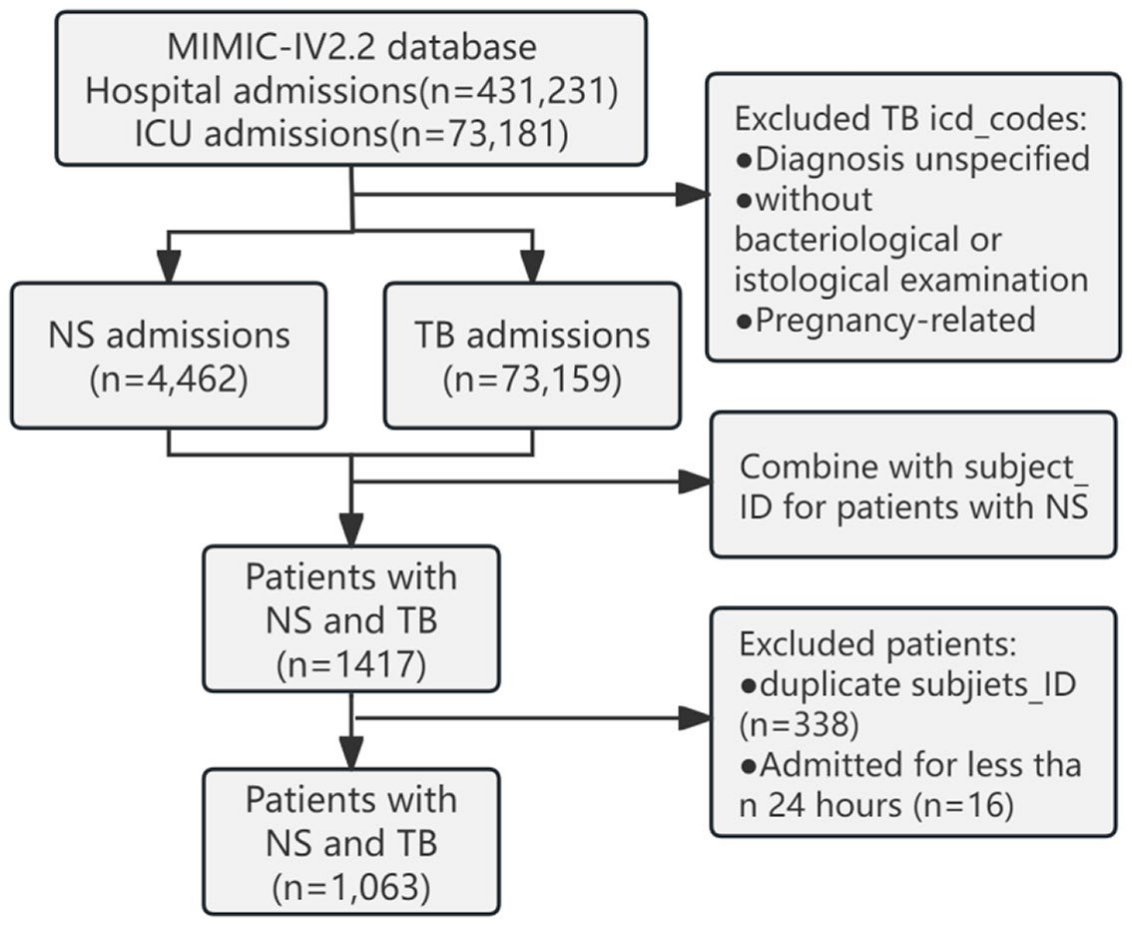
Schematic diagram of the study sample selection process. MIMIC, Medical Information Mart for Intensive Care; ICU, intensive care unit; NS, nephrotic syndrome; TB, tuberculosis.

The MIMIC-IV (v2.2) database documented 431,231 hospital and 73,181 ICU admissions. Based on the International Classification of Diseases, Ninth Revision (ICD-9) and the International Classification of Diseases, Tenth Revision (ICD-10) codes, 64 codes related to NS were extracted. TB cases were excluded if the diagnosis was unspecified, without bacteriological or histological examination, or if it was related to pregnancy; 298 codes were extracted. A total of 1,063 patients with NS admitted to the ICU with concurrent TB infection were identified. We retained demographic information from their first admission and extracted vital signs, laboratory indicators, and comorbidity information from their last hospital stay (Figure 1).

### Data extraction

NS with concurrent TB was the primary variable of interest with cumulative in-hospital mortality as the study endpoint. The extraction of potential confounding factors included demographics (age, gender), vital signs [average temperature (Temp), average respiration (Resp), average heart rate (HR), average blood pressure (Abp)], the maximum values of laboratory indicators [Hemoglobin (HGB), White Blood Cell Count (WBC), Glucose (Glu), Albumin (Alb), Blood Urea Nitrogen (Bun), Creatinine (Cr), Potassium, International Normalized Ratio (INR)], comorbidities [Myocardial Infarction (MI), Congestive Heart Failure (CHF), Peripheral Vascular Disease (PVD), Cerebrovascular Disease (CVD), Diabetes with Chronic Complications (Diabetes w/CC), Malignant Cancer (MC), Severe Liver Disease (SLD)], and the occurrence of Sepsis-3 and AKI stage within 7 days. Unfortunately, when extracting data for the types of drugs used, specifically rifampicin or rifapentine, from the prescription table in the database, no data were available. Data were extracted using PostgreSQL software (v13.13.1) and Navicat Premium software (version 16) by running Structured Query Language (SQL) queries.

### Management of missing data and outliers

During the data cleaning, it was discovered that there were missing values in the variables that represented potential confounding factors. Multiple imputation was employed to address this issue (20). Multiple imputation is a method based on repeated simulations for handling missing values, which can resolve complex missing data problems. A common concern when dealing with missing data is the acceptable proportion of missing data. The availability of predictors for missing data and/or variables related to the confounding factors associated with missing data may be a critical consideration. Even with a very high proportion of missing data (up to 90%), the fraction of missing information is more crucial than the proportion of missing data in terms of the precision of data analysis estimates. This approach helps reduce the bias in estimates (21).

### Statistical analysis

In this study, all data were statistically analyzed using the R statistical software package (R Foundation)^2^ and Fengrui statistical software version 1.9.The study employed a two-tailed test, with *p* < 0.05 considered statistically significant. Due to the presence of missing covariates in the collected data, with albumin and INR having a higher proportion of missing values, multiple imputations were performed to reduce bias. Continuous variables are expressed as mean ± standard deviation or median (interquartile range) depending on the situation. Categorical variables are presented as number (%). For the analysis of baseline characteristics, continuous variables were compared using the t-test or one-way ANOVA, while categorical variables were assessed using the chi-square test or Fisher’s exact test. Univariate logistic regression was used to analyze potential risk factors, and Least Absolute Shrinkage and Selection Operator (LASSO) regression cross-validation was employed to calculate the lambda values for the minimum cross-validation error and least standard error, selecting the most useful predictors from the derivation cohort. The diagnostic experiment The receiver operating characteristic (ROC) curve analysis model was used to model the predictors under two lambda values to assess their consistency and determine the determinants affecting prognosis. A Logistic prediction model was used to establish mortality risk nomograms, with the concordance index (C-index) measuring the predictive performance and discriminative ability of the nomogram, equivalent to the area under the ROC curve. Generally, a C-index ≥0.70 indicates a good fit. The model was evaluated using calibration curves with 500 bootstrap iterations and assessed on 200 randomly split data subsets with 500 iterations of 10-fold internal leave-one-out cross-validation and clinical decision curve analysis (DCA) to determine clinical utility and net benefit. Finally, nomograms were used to calculate the total score for each patient.

## Results

### Baseline clinical characteristics

The study included a total of 1,063 patients, with 362 females (34.1%) and 701 males (65.9%), with an average age of 66.3 ± 14.8 years. The rates of missing data for albumin and INR were 60.4 and 8.7%, respectively, whereas those for the other variables were below 5%. We employed logistic regression for multiple imputations, with five sets of imputations generated, and the third set was selected for the analysis. The cumulative in-hospital mortality rate of patients with NS and TB infections was 18.7% (Table 1). The Age, HR, Resp of the deceased patients were higher than those of the survivors, whereas Abp was lower (*p* < 0.001). Compared to the deceased, the surviving in-hospital patients had higher levels of HGB (*p* < 0.001) and Alb (*p* < 0.001), and a lower risk of Glu (*p* < 0.001). Among the deceased patients, the prevalence of sepsis-3 and AKI stage 7 days was 71.9 and 87.9%, respectively, which were significantly higher than those among the survivors, with a higher proportion of comorbidities, including statistically significant differences in congestive heart failure and severe liver disease.

**Table 1:** Baseline characteristics of NS with TB.

Variables	Total (*n* = 1,063)	Survival (*n* = 864)	Death (*n* = 199)	*P*
Age (yr)	66.3 ± 14.8	65.7 ± 14.9	69.0 ± 14.3	0.005
Gender, n (%)				0.969
Female	362 (34.1)	294 (34)	68 (34.2)	
Male	701 (65.9)	570 (66)	131 (65.8)	
Heart rate (beats/min)	85.1 ± 16.0	84.0 ± 14.9	89.9 ± 19.5	< 0.001
Average blood pressure (mmHg)	78.2 ± 11.9	79.5 ± 11.6	72.6 ± 11.4	< 0.001
Respiratory rate (bmp)	19.6 ± 4.1	19.2 ± 3.7	21.4 ± 5.1	< 0.001
Temperature (°C)	36.8 ± 0.4	36.8 ± 0.4	36.7 ± 0.7	0.001
Glucose (mol/L)	7.8 ± 2.9	7.6 ± 2.6	8.5 ± 3.8	< 0.001
Hemoglobin (g/L)	107.3 ± 20.7	108.1 ± 20.2	103.7 ± 22.3	0.007
White blood cell (*10^9^/L)	14.2 ± 11.5	13.7 ± 11.8	16.2 ± 10.1	0.007
Albumin (g/L)	31.8 ± 6.4	32.6 ± 6.2	28.5 ± 6.3	< 0.001
Blood urea nitrogen (mg/dL)	36.0 ± 27.2	32.8 ± 24.4	49.8 ± 33.7	< 0.001
Creatinine (mg/dL)	2.3 ± 2.4	2.1 ± 2.4	3.0 ± 1.9	< 0.001
Potassium (mEq/L)	4.7 ± 0.9	4.7 ± 0.9	4.9 ± 0.9	0.007
International normalized ratio	1.7 ± 1.2	1.6 ± 1.0	2.3 ± 2.0	< 0.001
Myocardial infarct, n (%)				0.331
No	746 (70.2)	612 (70.8)	134 (67.3)	
Yes	317 (29.8)	252 (29.2)	65 (32.7)	
Congestive heart failure, n (%)				0.007
No	498 (46.8)	422 (48.8)	76 (38.2)	
Yes	565 (53.2)	442 (51.2)	123 (61.8)	
Peripheral vascular disease, n (%)				0.648
No	672 (63.2)	549 (63.5)	123 (61.8)	
Yes	391 (36.8)	315 (36.5)	76 (38.2)	
Cerebrovascular disease, n (%)				0.879
No	917 (86.3)	746 (86.3)	171 (85.9)	
Yes	146 (13.7)	118 (13.7)	28 (14.1)	
Diabetes with/CC, n (%)				0.138
No	867 (81.6)	712 (82.4)	155 (77.9)	
Yes	196 (18.4)	152 (17.6)	44 (22.1)	
Malignant cancer, n (%)				0.323
No	880 (82.8)	720 (83.3)	160 (80.4)	
Yes	183 (17.2)	144 (16.7)	39 (19.6)	
Severe liver disease, n (%)				0.022
No	1,007 (94.7)	825 (95.5)	182 (91.5)	
Yes	56 (5.3)	39 (4.5)	17 (8.5)	
Sepsis, n (%)				< 0.001
No	564 (53.1)	508 (58.8)	56 (28.1)	
Yes	499 (46.9)	356 (41.2)	143 (71.9)	
AKI stage 7-day, n (%)				< 0.001
Stage 0	364 (34.2)	340 (39.4)	24 (12.1)	
Stage 1	173 (16.3)	150 (17.4)	23 (11.6)	
Stage 2	289 (27.2)	240 (27.8)	49 (24.6)	
Stage 3	237 (22.3)	134 (15.5)	103 (51.8)	

### Univariate survival analysis

Univariate logistic regression analysis showed that for each unit increase in Resp and HR, the risk of in-hospital mortality increased by 13 and 2%, respectively. For each 1 mmHg increase in Abp, the risk of in-hospital mortality decreased by 6% (OR = 0.94, 95% CI: 0.93–0.96), with a *p*-value of less than 0.001 (Table 2). The odds ratios (OR) for comorbidities were all greater than 1, with patients with sepsis-3 having an OR of 3.64 (2.6 ~ 5.11), CHF 1.55 (1.13 ~ 2.12), and SLD 1.98 (1.09 ~ 3.57). For patients with AKI stage 7-day, the higher the stage level, the higher the risk of in-hospital mortality, with stage 3 having an OR of 10.89 (6.69 ~ 17.72) (*p* < 0.001). For each unit increase in the INR, the risk of death increased by 45%. The OR value for serum albumin was 0.9 (0.88 ~ 0.93), *p* < 0.001, and for each unit increase in Glu, Bun, Cr, and potassium, the risk of death increases by 9, 2, 14, and 24%, respectively, showing a significant statistical association with the risk of in-hospital mortality. Sex was not associated with the risk of death.

**Table 2:** Univariate logistic regression analysis of hospital incidence.

Variable	OR_95CI	*P*_value
Age	1.02 (1 ~ 1.03)	0.005
Male	0.99 (0.72 ~ 1.38)	0.969
Heart rate (beats/min)	1.02 (1.01 ~ 1.03)	<0.001
Average blood pressure (mmHg)	0.94 (0.93 ~ 0.96)	<0.001
Resp (bmp)	1.13 (1.09 ~ 1.17)	<0.001
Temp (°C)	0.55 (0.38 ~ 0.8)	0.001
Glucose (mol/L)	1.09 (1.04 ~ 1.15)	<0.001
Hemoglobin (g/L)	0.99 (0.98 ~ 1)	0.007
White blood cell (*109/L)	1.02 (1 ~ 1.03)	0.027
Albumin (g/L)	0.9 (0.88 ~ 0.93)	<0.001
Blood urea nitrogen (mg/dL)	1.02 (1.01 ~ 1.02)	<0.001
Creatinine (mg/dL)	1.14 (1.07 ~ 1.2)	<0.001
Potassium (mEq/L)	1.24 (1.06 ~ 1.46)	0.008
International normalized ratio	1.45 (1.28 ~ 1.65)	<0.001
Myocardial infarct	1.18 (0.85 ~ 1.64)	0.331
Congestive heart failure	1.55 (1.13 ~ 2.12)	0.007
Peripheral vascular disease	1.08 (0.78 ~ 1.48)	0.648
Cerebrovascular disease	1.04 (0.66 ~ 1.61)	0.879
Diabetes with/CC	1.33 (0.91 ~ 1.94)	0.139
Malignant cancer	1.22 (0.82 ~ 1.81)	0.324
Severe liver disease	1.98 (1.09 ~ 3.57)	0.024
Sepsis	3.64 (2.6 ~ 5.11)	<0.001
AKI stage 7-day		
Stage 1	2.17 (1.19 ~ 3.97)	0.012
Stage 2	2.89 (1.73 ~ 4.84)	<0.001
Stage 3	10.89 (6.69 ~ 17.72)	<0.001

### Model specifications and predictive factors for progression

**Figure 2:**
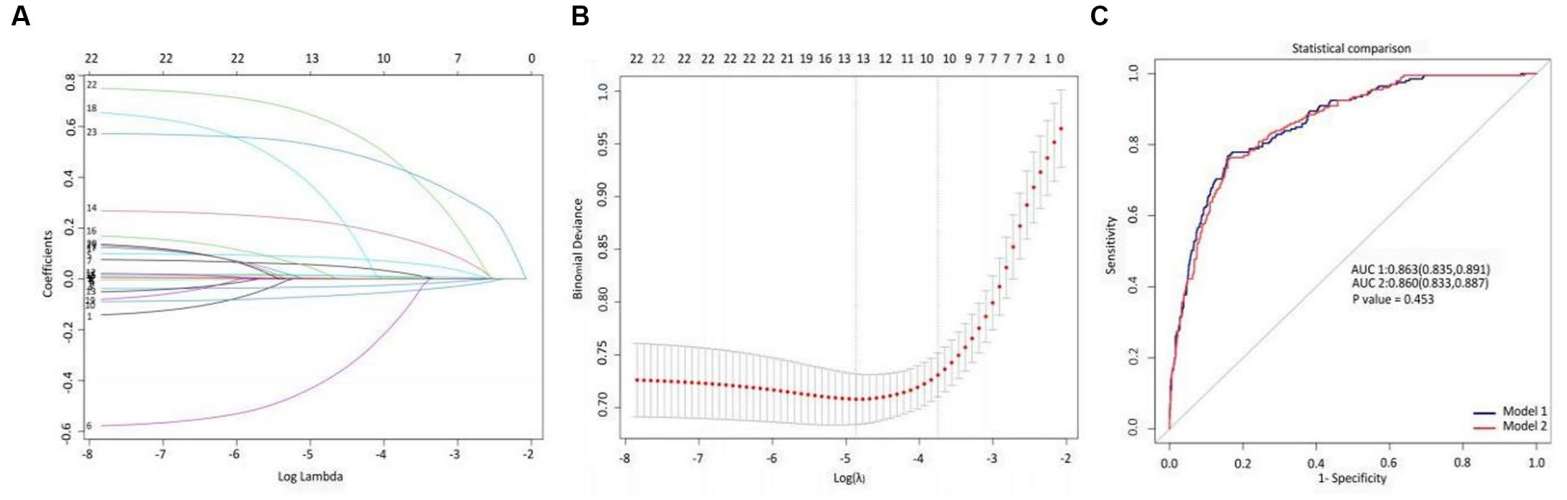
LASSO variable selection and diagnostic test ROC model evaluation. **(A)** Overview of LASSO coefficients for 23 clinical and pathological features, **(B)** deviation curve of LASSO cross-validation, and **(C)** ROC analysis evaluation of diagnostic experiments based on critical variables selected by lambda.min and lambda.lsewith a *p*-value of 0.453.

Using LASSO regression, we identified the minimum lambda value for cross-validation error as 0.008 (lambda.min) and the lambda value for the least standard error (LSE) as 0.024 (lambda.lse). From these 23 variables, we selected 13 and 10 potential determinants for the two groups, respectively (Figures 2A,B). These groups of variables were included in the ROC diagnostic analysis. The results showed that the area under the curve (AUC) for the former was 0.863 (0.835–0.891) and that for the latter was 0.860 (0.833–0.887), with a *p*-value of 0.450 (Figure 2C), indicating no statistical significance. To simplify the model for practical clinical use, based on the lambda value of 0.024 for lambda.lse and the evaluation of the ROC diagnostic analysis model, we included HR, Abp, Resp., INR, Alb, Bun, Glu, Sepsis-3, AKI stage 7 days, and CVD in the construction of a nomogram.

### Nomogram and model performance

**Figure 3:**
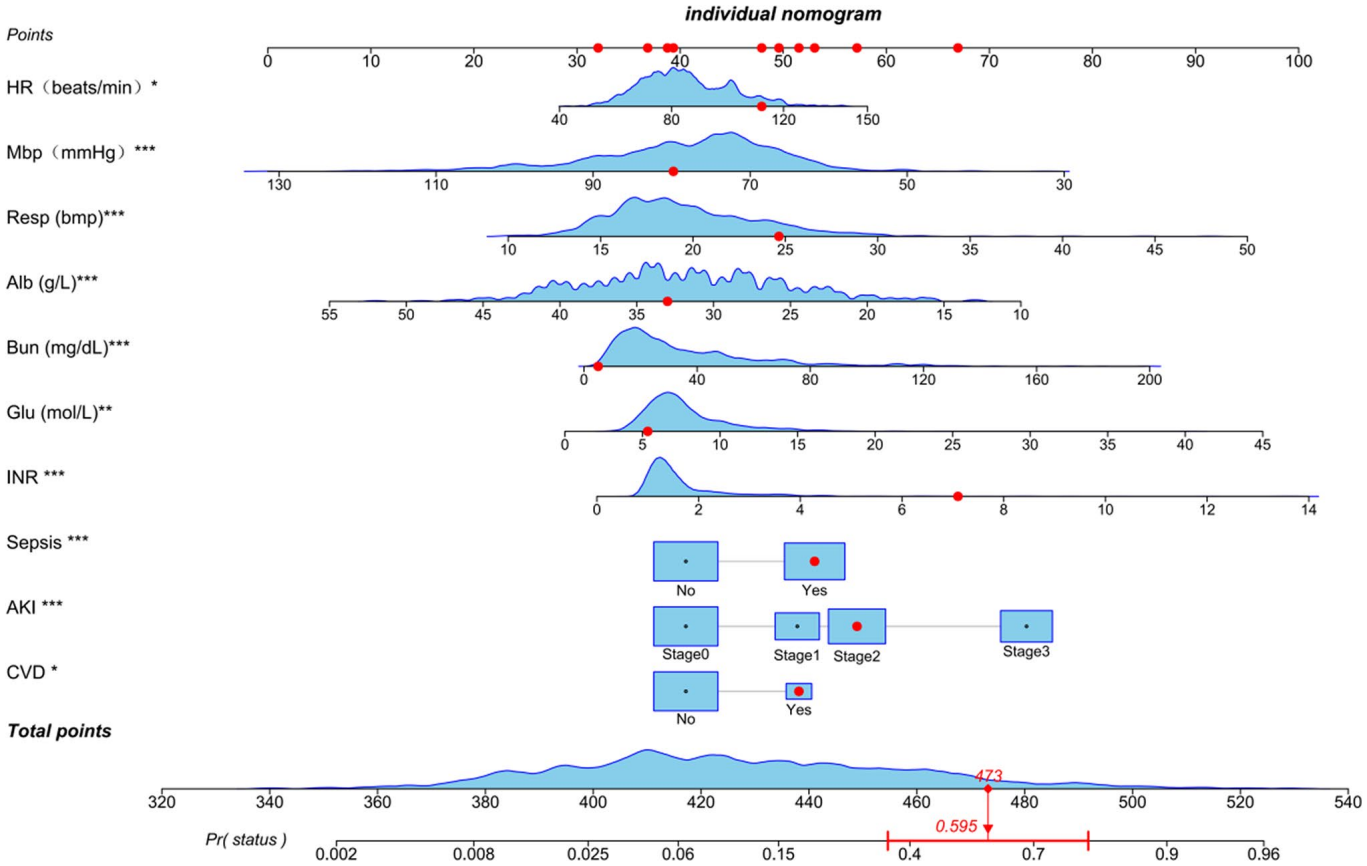
Nomogram for estimating mortality risk: Each red dot corresponds to a score point for a specific variable, resulting in an overall score of 479 and a 59.5% probability of death. The levels of statistical significance are indicated by the asterisks, with *p < 0.05, **p < 0.01, and ***p < 0.001.

**Figure 4:**
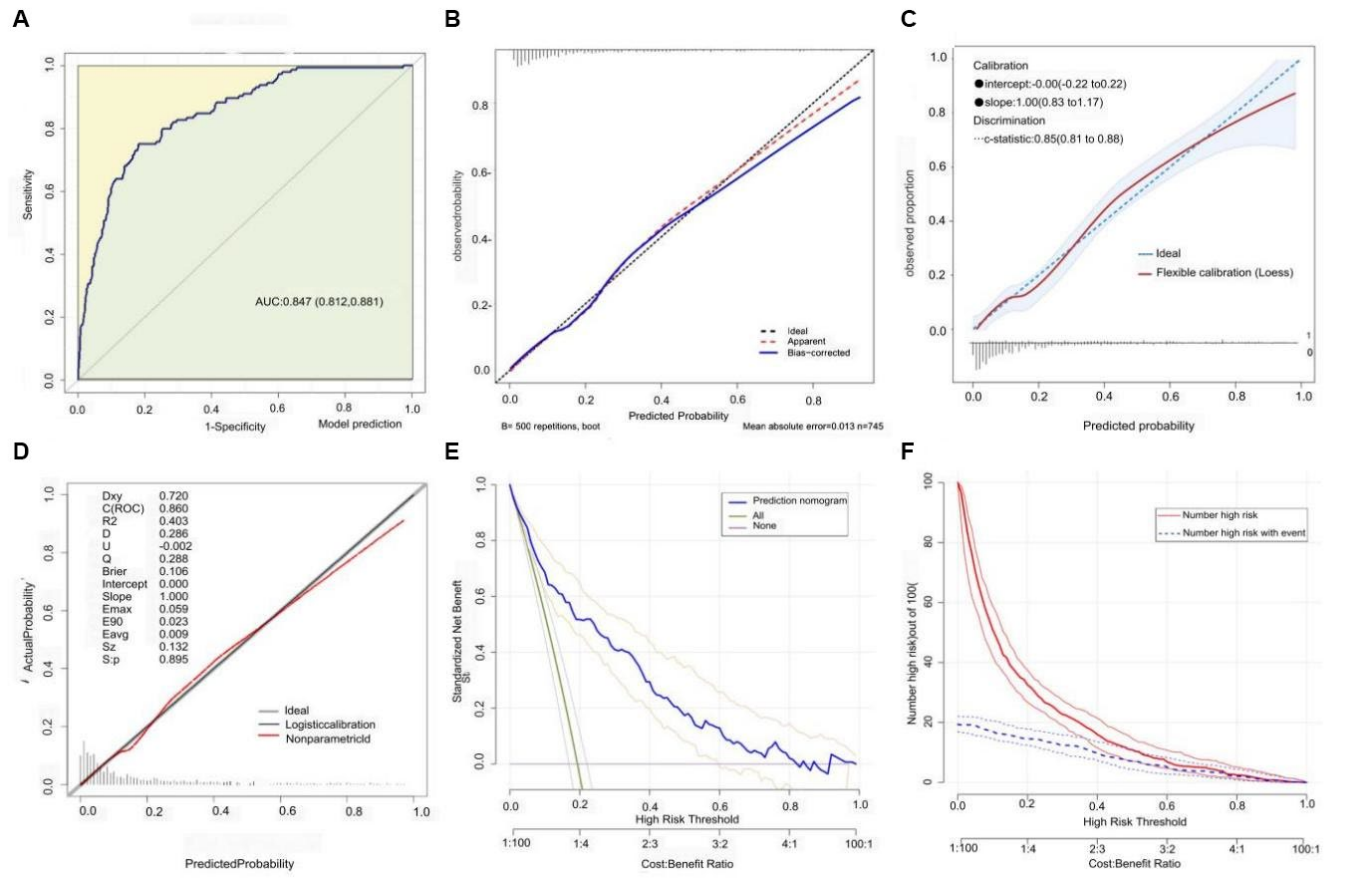
Model discrimination and calibration evaluation. **(A)** ROC curve with an AUC of 0.847 [95% CI: 0.812–0.881]. **(B)** Flexible calibration with a slope of 1.0 (0.83 ~ 1.17). **(C)** Bootstrap cross-validation (MAE = 0.013). **(D)** Internal leave-one-out cross-validation post-calibration C-index of 0.860 and calibration slope of 1.0. **(E)** Decision curve analysis. **(F)** Clinical impact curve analysis.

Based on the previously mentioned 10 determinants, we created a nomogram where the sum of the corresponding points for each factor indicated that a higher total score was associated with a worse prognosis (Figure 3). Figure 4A shows the ROC curve evaluation of the nomogram with an AUC of 0.847 (0.812 ~ 0.881) (Figure 4A). Bootstrap resampling with 500 repetitions for cross-validation revealed that the mean absolute error (MAE) between the model predictions and the actual values was 0.013 (Figure 4B). A Flexible Calibration (Loess) intercept of −0.00 (−0.22–0.22), and a calibration slope of 1.00 (0.83–1.17) (Figure 4C). Leave-one-out internal cross-validation was used to assess the model, with a corrected C-index of 0.860 and calibration slope of 1.0 (Figure 4D). Figure 4E demonstrates that DCA indicated that patients could benefit from this model when the threshold was between 0.1 and 0.81 (Figure 4E). With a risk threshold above 0.5, the predictive model closely aligned with the actual risk curve (Figure 4F).

## Discussion

In this retrospective cohort study, we investigated the data of patients with NS complicated by TB admitted to the ICU from the MIMIC-IV v2.2 database. We also proposed a risk prediction model for key factors affecting in-hospital mortality. Notably, our model exhibited a good performance and calibration. The c-index evaluated using the ROC curve was 0.847 (0.812–0.881), indicating that the model had an excellent discriminative ability to accurately differentiate between survival and death outcomes. The slopes of both the flexibility calibration and cross-validation curves were equal to 1, and the MAE between the predicted and actual values was 0.013, proving the high accuracy and reliability of our model for predicting in-hospital mortality in patients with NS complicated by TB. In the process of building a predictive model for in-hospital mortality based on NS complicated by TB infection, we observed an in-hospital mortality rate of 18.7%. Through LASSO regression and diagnostic ROC analysis, we identified 10 key prognostic factors: HR, Abp, Resp., Alb, Bun, INR, Glu, and the occurrence of Sepsis-3, AKI, and CVD. Unfortunately, we were unable to extract effective information on TB drugs and steroid use from the database, which prevented us from developing a more comprehensive model from a treatment perspective that reflected the current dilemmas in treating NS complicated by TB.

Vital signs are indicators of the basic state of life of the body, which can undergo compensatory adjustments due to illness. However, once decompensation occurs, the patient’s condition may worsen significantly. A study on the prediction model for in-hospital mortality among patients with moderate thrombocytopenia showed that each unit increase in heart rate and respiratory rate increased the risk of death by 2.6 and 11.6%, respectively, while each 1 mmHg increase in blood pressure reduced the risk of death by 4.7% (22). Heart and respiratory rates were significantly and linearly correlated with the 30-day mortality rate of critically ill patients with coronavirus disease (23). Univariate analysis showed that each unit increase in the HR and Resp increased the risk of in-hospital mortality by 2 and 13%, respectively. Meanwhile, an increase in Abp could reduce the risk of in-hospital death, with each 1 mmHg increase decreasing the risk by 6%. These results are consistent with those reported in literature, underscoring the importance of vital sign stability in disease recovery, particularly in critically ill patients.

NS leads to a reduction in immunity owing to the loss of a large amount of immunoglobulins and complement, along with a decrease in regulatory T cells (Tregs), which further diminish as the disease progresses (24–26). *Mycobacterium tuberculosis* can directly damage macrophages (27) and gradually deplete natural killer (NK) cell subsets (CD3-CD7 + GZMB+) (28), impairing the immune system. This compounded immunopathological change may lead to disease progression and a higher likelihood of concurrent mixed microbial infections, resulting in sepsis-3. Sepsis-3 can directly or indirectly harm the function of immune cells (29), creating a vicious cycle. This may be related to the high prevalence of sepsis-3 (46.9%) among the ICU patients in this study. Tuberculosis infection can also lead to tuberculosis-related sepsis-3, with a 30-day survival rate of only 33% for such patients (30), who are often overlooked and not promptly treated. In 2017, sepsis-3 deaths accounted for 19.7% of global mortality (31). In our study, the prevalence of sepsis-3 among deceased patients was as high as 71.9%, with the risk of in-hospital death increasing by 2.64 times, explaining why sepsis-3 was selected as a key variable in our predictive model through LASSO regression. Additionally, an important complication that cannot be ignored is AKI, which significantly increases the risk of death (32). AKI may be related to sepsis (33) but is also closely associated with NS and TB (34–37). Our study found that the prevalence of AKI at 7 days was 65.8%, with a prevalence of 87.9% among deceased in-hospital patients. The risk of death increased by 9.89 times for AKI stage 3, making AKI a significant indicator of mortality risk. In addition to CVD, other comorbidities were not included in the model, but were important risk factors for in-hospital death in the univariate analysis, especially CHF, with a prevalence rate of 53.2% and as high as 61.8% in the death group.

Furthermore, NS and TB can lead to reduced appetite, decreased nutritional intake, and increased metabolic rate, making control of the condition challenging and hypoalbuminemia inevitable. Our findings showed that, in the in-hospital death group, the albumin level was 28. ± 6.3 g/L, with each unit increase in albumin level reducing the risk of death by 7%. Low serum albumin levels (<34 g/L) can independently predict the 30-day mortality rate in adult patients in the medical emergency department of a Swiss tertiary care center, with an area under the ROC curve of 0.77 (38), and is highly correlated with cardiovascular events and mortality risk (39, 40). INR, Glu, and Bun are laboratory parameters of interest in LASSO regression, with each unit change in these indicators causing 45, 9, and 2% fluctuations in death risk, respectively. INR reflects coagulation system function; NS glomerular lesions lead to the loss of low-molecular-weight anticoagulant proteins, whereas high-molecular-weight procoagulant proteins (HMWPP) are retained in the plasma (41, 42). Clinical data show that the more severe the NS (41), the higher the incidence of thrombus formation. However, anticoagulant treatment for NS, renal replacement therapy for AKI, and the loss of coagulant proteins can increase the risk of bleeding. Our findings indicated an average INR of 2.3 among deceased patients, suggesting an increased risk of bleeding. Elevated Bun levels are associated with renal function decline and protein catabolism and represent an independent risk factor for prognosis in critically ill patients, especially those with cardiac diseases (43, 44). Acute stress during the disease may make short-term moderate hyperglycemia beneficial, ensuring glucose supply to immune cells (45), activating anti-apoptotic pathways, and benefiting angiogenesis (46). However, significant and persistent hyperglycemia is associated with adverse outcomes and has been identified as an independent predictor of in-hospital mortality (47).

In this study, we developed a prognostic model using the above 10 key factors, which were evaluated using the C-index, multiple forms of calibration curves, and discussion analysis, demonstrating good performance, accuracy, and scientific validity. Further analysis with clinical decision and impact curves indicated that patients could benefit from this model, with a risk threshold of 0.02 and 0.81. Moreover, the model’s ability to identify risk significantly improved when the risk threshold exceeded 0.5. Our nomogram accurately predicted a 59.5% risk of in-hospital mortality in patients with a total score of 473, demonstrating good applicability. However, our study has some limitations, including the inability to extract essential treatment information for NS and TB, owing to the retrospective nature of the study and the impact of missing key confounding variables. We used multiple imputations to reduce the impact of missing confounders and to avoid bias, thus stabilizing our results. The lack of external validation indicates that further research is needed to validate our predictive model with a larger sample size over extended periods to draw further conclusions. Furthermore, this study was a single-center clinical study involving patients with severe conditions and a relatively older average age. Therefore, further research is needed to confirm the prognosis of younger patients and those from different regions.

In summary, our clinical prediction nomogram demonstrated a good predictive ability for the prognosis of patients with NS complicated by TB, aiding healthcare professionals in initial risk assessment, prognosis judgment, and clinical decision-making. Patients with NS and TB represent a special group that requires further clinical research on treatment strategies and prognoses. Despite the need for more comprehensive predictive models, collaboration between nephrologists and tuberculosis specialists, as highlighted by our two unpublished successful treatment cases, is essential.

## Data availability statement

Publicly available datasets were analyzed in this study. This data can be found at: the Medical Information Mart for Intensive Care IV version 2.2 [MIMIC-IV (v2.2)] database.

## Author contributions

JH: Funding acquisition, Writing – original draft, Writing – review & editing. SD: Conceptualization, Data curation, Methodology, Writing – original draft, Writing – review & editing. NS: Formal analysis, Supervision, Validation, Writing – original draft, Writing – review & editing. ZY: Conceptualization, Resources, Writing – original draft, Writing – review & editing. JL: Resources, Visualization, Writing – original draft, Writing – review & editing. YJ: Software, Writing – original draft, Writing – review & editing. LZ: Software, Visualization, Writing – original draft, Writing – review & editing.

## Footnotes

^1^ physionet.org

^2^ http://www.R-project.org
